# Effects of introducing eels on the yields and availability of fertilizer nitrogen in an integrated rice–crayfish system

**DOI:** 10.1038/s41598-020-71884-0

**Published:** 2020-09-09

**Authors:** Weiwei Lv, Quan Yuan, Weiguang Lv, Wenzong Zhou

**Affiliations:** Eco-Environmental Protection Research Institute, Shanghai Academy of Agricultural Sciences, Shanghai, 201403 China

**Keywords:** Biogeochemistry, Climate sciences

## Abstract

Recently, many new rice–fish co-culture models have been developed to increase economic and ecological benefits. In this study, we added eels (*Monopterus albus*) to a rice–crayfish system and conducted a 3-year field investigation to compare the yields and availability of fertilizer N among groups with a low density of eels, high density of eels and no eels. We performed a mesocosm experiment and used an isotope tracer technique to detect the fate of fertilizer N. The results showed that the rice yields significantly improved after the introduction of the eels. However, the introduction of a high density of eels significantly limited the crayfish yield, increased water N and N_2_O emissions and decreased soil N content. The mesocosm experiment suggested that the use efficiency of fertilizer N was significantly increased after the introduction of the eels. The fertilizer N used by rice was significantly higher in rice–crayfish–eel system than in rice–crayfish system. This study indicated that the introduction of eels may be a good practice for improving yields and availability of fertilizer N in a rice–crayfish system.

## Introduction

In recent years, a substantial increase in integrated rice–fish culture (IRFC) has been observed in China. By the end of 2010, IRFC covers an area of 1.33 × 10^6^ ha, which accounts for 4.48% of the total rice planting area in China^1^. The primary concept of IRFC is to culture aquatic animals, e.g. fish, shrimp, crabs and soft shell turtles, in limited paddy space^2,3^. When compared with monoculture of rice and fish, IRFC has advantages, such as optimizing land resources, saving labour inputs and facilitating field management^4^. IRFC is considered an effective agriculture mode that can simultaneously provide food security and conserve the environment because almost no pesticides or herbicides are used during rice production^5^. Moreover, IRFC can increase fertilizer availability and decrease fertilizer application^6–8^.

Nitrogen (N) fertilizer provides an essential nutrient for rice cultivation. In IRFC, the availability of soil or fertilizer N can be enhanced through the complementary use of N by rice and fish^9^. The activities of aquatic animals can increase N release from the soil and N uptake by rice plants^10–13^. Fish excrement and effluents also have a fertilizing effect, which increases the amount of nutrients available to the rice crops^14^. Excess N temporarily retained in rice fields can be transmitted through the food chain by aquatic animals^15^. Therefore, when compared with rice monoculture, IRFC can greatly reduce N loss to the environment.

Integrated rice–crayfish (*Procambarus clarkii*) culture (IRCC) is one of the most popular IRFCs in China. Currently, the total area under IRCC in China is 6 × 10^5^ ha, and 1.2 × 10^6^ t crayfish are produced from paddy fields^16^. The co-culture of rice and crayfish can improve the soil carbon pool and microbial community structure^17^. Crayfish activities can contribute to a high rice yield from paddy fields^18^. However, the explosive increase in crayfish production has greatly limited the economic benefits of IRCC in the past few years. Moreover, crayfish farming relies too much on artificial diets, which may cause water pollution and environmental damage. To increase income and ecological health, many farmers have tried to introduce new species into the rice–crayfish (RC) system to develop new IRFCs with more complex species combinations, such as rice–crayfish–eel and rice–crayfish–turtle co-cultures^19^.

In China, the Asian swamp eel (*Monopterus albus*) is an indigenous species with a high economic value. The eels can adapt to the complex environment of rice fields, and they are considered as an ideal species for rice paddy farming^20^. The introduction of eels to the RC system can improve spatial efficiency, prolong the food chain and increase biodiversity^21^. Many scholars have detected N availability in the RC system. Previous studies have suggested that IRCC does not significantly increase the N uptake in rice grains, roots and straw when compared with rice monoculture^22^. Moreover, the co-culture of rice and crayfish may cause more N loss in the form of N_2_O from the paddy ecosystem^23^. However, there is limited information on the effects of eel or eel–crayfish disturbance on the N cycle in paddy fields.

In this study, we investigated a rice–crayfish–eel (RCE) system continuously for 3 years. Meanwhile, we performed a mesocosm experiment and used a stable isotope (^15^N) tracer technique. The aims of this study were to (1) analyse the effects of eel introduction on the yields of rice and crayfish and (2) detect the availability of fertilizer N in the RCE system.

## Results

### Yields in field investigation

Table 1 shows the average yields and total N content of rice, crayfish and eels. The rice yields decreased significantly in the control paddies during the investigation (*P* < 0.05, Table S1). However, no significant changes were observed in the yields of rice, crayfish and eels in the LD and HD groups (*P* > 0.05, Table S1). From 2018 to 2019, the rice yields were significantly higher in the LD and HD groups than in the control group (*P* < 0.05, Table S1). However, the crayfish yields in the HD group were significantly lower than that in the LD and control paddies (*P* < 0.05, Table S1). The eel yields were significant higher in the HD group than in the LD group (*P* < 0.05, Table S1). The total N content of rice, crayfish and eels had similar spatial and temporal trends with the rice yields.

**Table 1:** Yields and total N content of rice, crayfish and eels in different co-culture systems: rice–fish culture (C), rice–crayfish–eel with a low density of eels (LD) and rice–crayfish–eel with a high density of eels (HD).

		Yield (t ha^−1^)			Total N content (kg ha^−1^)		
		2017	2018	2019	2017	2018	2019
Rice	Control	5.7 ± 0.2^A^	4.7 ± 0.2^Bb^	4.4 ± 0.5^Bb^	75.0 ± 6.2^b^	70.5 ± 3.7^b^	64.0 ± 6.2^b^
	LD	6.3 ± 0.1	5.9 ± 0.4^a^	6.2 ± 0.1^a^	92.4 ± 2.5^a^	93.2 ± 2.1^a^	90.5 ± 1.3^a^
	HD	6.1 ± 0.5	6.2 ± 0.3^a^	6.6 ± 0.3^a^	95.0 ± 8.6^a^	89.0 ± 6.1^a^	86.1 ± 11.7^a^
Crayfish	Control	0.8 ± 0.1^a^	0.9 ± 0.2^a^	0.7 ± 0.1^a^	17.2 ± 1.2^a^	16.5 ± 2.7^a^	15.8 ± 1.3^a^
	LD	1.0 ± 0.1^a^	0.9 ± 0.2^a^	0.8 ± 0.1^a^	16.7 ± 2.4^a^	15.7 ± 3.1^a^	15.1 ± 2.7^a^
	HD	0.4 ± 0.1^b^	0.3 ± 0.1^b^	0.4 ± 0.1^b^	13.0 ± 2.1^b^	11.2 ± 2.4^b^	8.9 ± 1.9^b^
Eel	Control	0^c^	0^c^	0^c^	0^b^	0^c^	0^b^
	LD	0.2 ± 0.04^b^	0.2 ± 0.1^b^	0.2 ± 0.1^b^	3.3 ± 0.6^b^	3.5 ± 0.7^b^	4.1 ± 0.7^b^
	HD	0.5 ± 0.1^a^	0.6 ± 0.1^a^	0.7 ± 0.1^a^	8.7 ± 1.4^a^	10.3 ± 1.1^a^	12.1 ± 1.7^a^

The capital letters represent a significant difference among the three investigated years (*P* < 0.05). The lowercase letters represent a significant difference among the three co-culture systems (*P* < 0.05).

### Total N content in the water and soils

**Figure 1:**
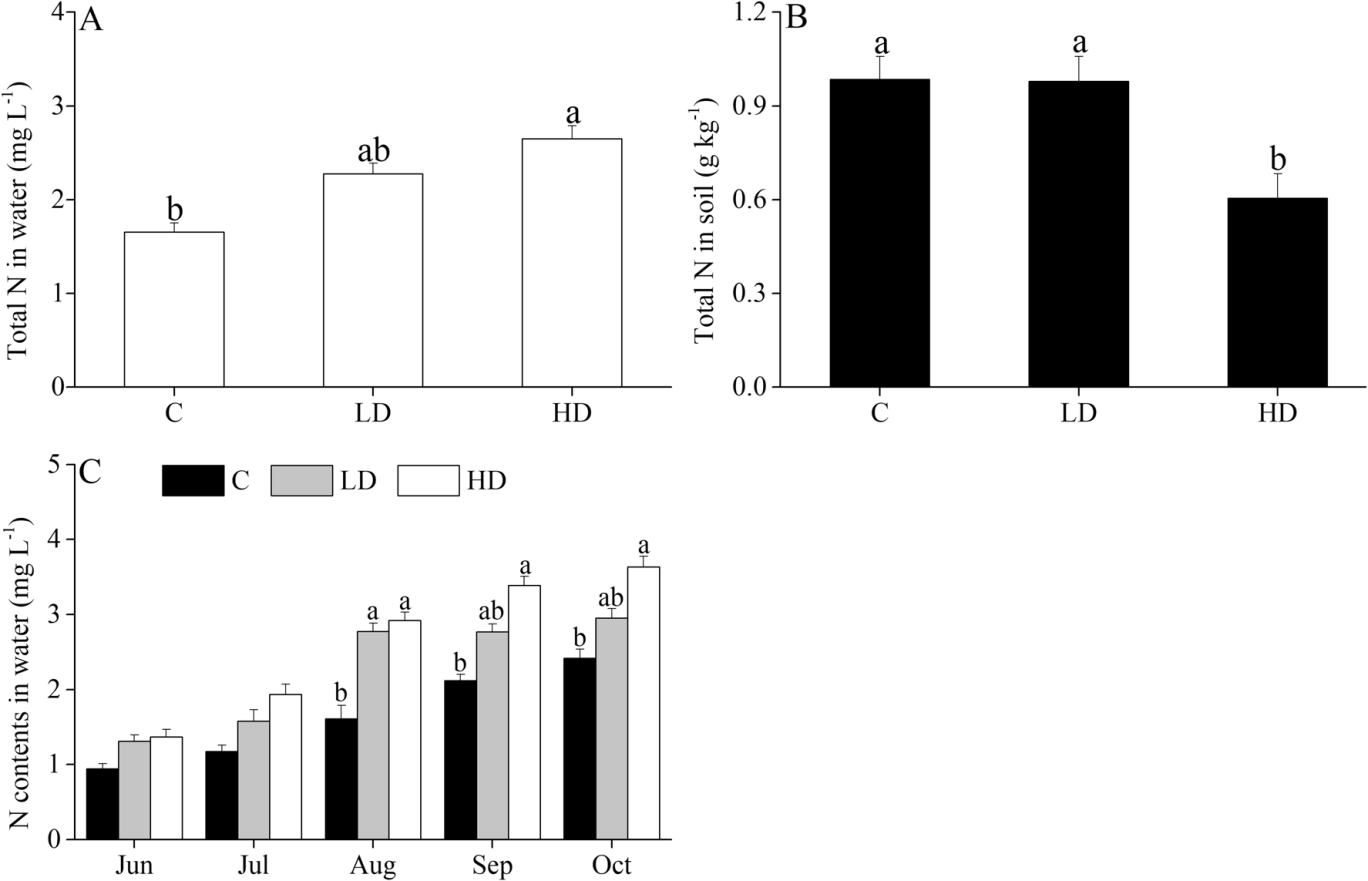
Average values of total N in the water (**A**) and soils (**B**) and monthly changes in total N in the water (**C**) of the different co-culture systems: rice–fish culture (C), rice–crayfish–eel with a low density of eels (LD) and rice–crayfish–eel with a high density of eels (HD). The lowercase letters represent a significant difference among the three co-culture systems (*P* < 0.05).

Figure 1 shows the changes in water and soil N during the 3-year investigation. No significant changes in water and soil N were observed during the 3 years (*P* > 0.05, Table S2). The average content of total N in the water was significantly higher in the HD group than in the control paddy (*P* < 0.05, Table S2). From August to October, the total N content in the water was significantly higher in the HD group than in the control group (*P* < 0.05, Table S2). However, no significant differences in water N were observed among the three groups from June to July (*P* > 0.05, Table S2). In contrast, the soil N was significantly lower in the HD group than in the control and LD paddies (*P* < 0.05, Table S2).

### N_2_O emission and NH_3_ volatilization

**Figure 2:**
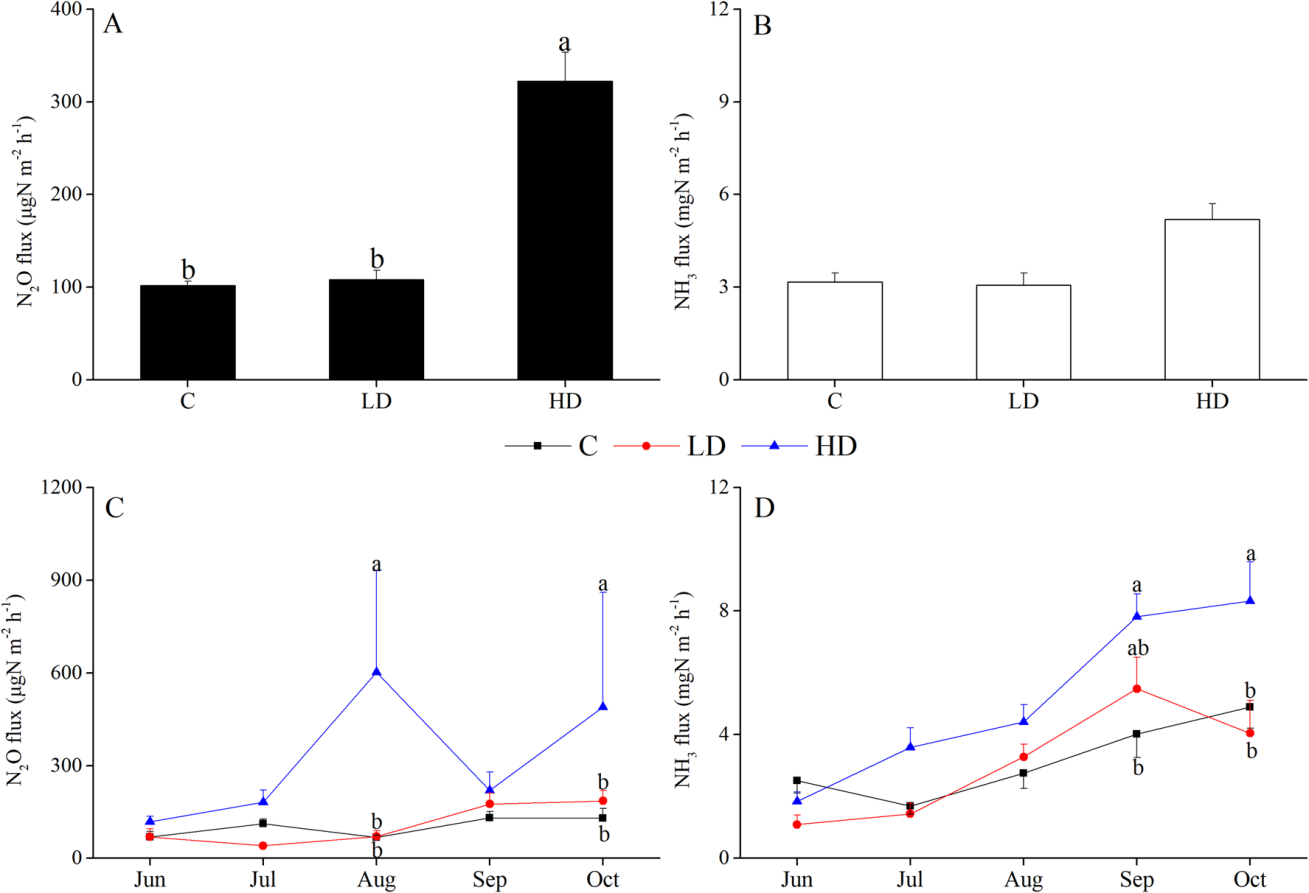
Average values of N_2_O emission (**A**) and NH_3_ volatilization (**B**) and monthly changes in N_2_O (**C**) and NH_3_ (**D**) in the different co-culture systems: rice–fish culture (C), rice–crayfish–eel with a low density of eels (LD) and rice–crayfish–eel with a high density of eels (HD). The lowercase letters represent a significant difference among the three co-culture systems (*P* < 0.05).

Figure 2 shows the variations in N_2_O emission and NH_3_ volatilization from the three rice–fish groups. The average N_2_O flux was significantly higher in the HD group than in the other two groups (*P* < 0.05, Table S3). In August and October, the N_2_O flux was significantly higher in HD group than in LD and control groups (*P* < 0.05, Table S3). However, no significant differences in N_2_O flux were observed in the other 3 months among three groups (*P* > 0.05, Table S3).

No significant differences in average NH_3_ volatilization were observed among the three groups (*P* > 0.05, Table S3). The NH_3_ flux was significantly higher in HD group than in control group (*P* < 0.05, Table S3) from September to October. However, there were no significant differences in NH_3_ flux among three groups from June to August (*P* > 0.05, Table S3).

### Fate of fertilizer N

**Figure 3:**
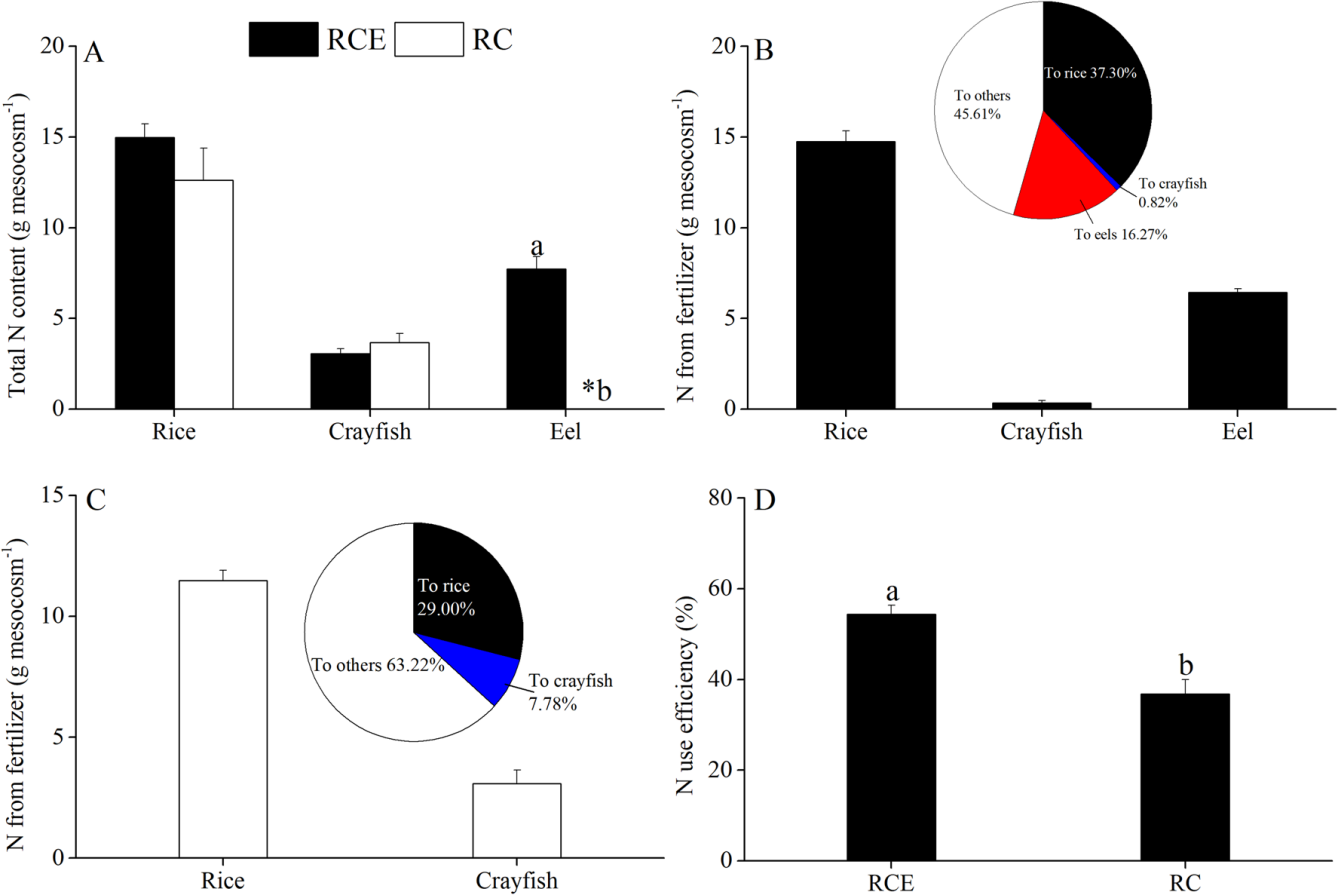
The total N content of the organisms (**A**), fate of fertilizer N (**B**,**C**) and N use efficiency (**D**) in the mesocosm experiment. RCE and RC represent rice–crayfish–eel and rice–crayfish systems, respectively. *indicates that no samples were collected from the mesocosm. The lowercase letters represent a significant difference among the different species (*P* < 0.05). The capital letters represent a significant difference between REC and RC (*P* < 0.05).

Figure 3 shows the fate of fertilizer N in the RCE and RC systems. No significant differences in the total N content of rice (*P* = 0.228) and crayfish (*P* = 0.334) were observed between the RCE and RC systems after the mesocosm experiment. The use efficiency of fertilizer N was significantly higher in RCE (54.39%) than in RC (36.78%; *P* = 0.009). The proportion of fertilizer N used by crayfish was significantly higher in RC (7.78%) than in RCE (0.82%; *P* = 0.003). In contrast, the proportion of fertilizer N used by rice was significantly lower in RC (29.00%) than in RCE (37.30%; *P* = 0.048). In the RCE system, about 16.27% of N was transferred to the eels from the fertilizer.

## Discussion

This study demonstrated that the introduction of eels at a low density significantly increased rice yields in the RC system. Moreover, no significant changes in rice and crayfish yields of the LD group occurred during the investigation. The results suggest that eels and crayfish can be bred simultaneously in paddies, despite their predation relationship. Moreover, the rice yields may be enhanced by multispecies complex rearing in the rice field. This viewpoint was also demonstrated by other scholars^24,25^. Lin and Wu found that the rice yields were significantly higher in the rice–frog–fish system than in rice–fish co-culture and rice monoculture^25^. In the rice fields, the eels prefer high temperatures and are usually active in the rice-growing platform. In contrast, the crayfish are aquatic animals that prefer shady areas and usually live at the bottom of ditches. The living spaces of the eels and crayfish do not completely coincide. The addition of eels can restrict the activities of crayfish in the rice-planting platform, thus reducing the destruction of rice roots by the crayfish. Therefore, an appropriate number of eels may promote the sustainable development of the RCE system.

This study also suggests that the crayfish yields were significantly suppressed by a high density of eels, although the rice yields were significantly increased. Previous studies have shown that bioturbation by eels is beneficial for maintaining the ecological security of paddies and rice yields because they prey on insect pests^26^. However, the eels also feed on benthic animals and fishes. The crayfish, especially the juvenile ones, may become the major food source of eels in the RCE system^27^. In rice fields, cultivation of crayfish would mainly require self-propagation and self-breeding; thus, the crayfish juveniles would probably be heavily preyed on by the eels, leading to an inevitable population degradation. In addition, we found that the concentration of water N in high-density group was significantly enhanced. However, the variation of ammonia can alter the duration and intensity of agonistic interactions in the crayfish^28^. Therefore, the decline in crayfish populations could also be caused by cannibalism.

In this study, the introduction of eels considerably increased water N and decreased soil N. Moreover, previous studies have suggested that the process of N release is affected by many abiotic and biotic factors, e.g. temperature, mobility and rearing density^29^. We found that the water N content was significantly higher in the HD group than in the control group from August to October. In addition, bioturbation by the eels in the rice platform may have loosened the soil structure, thus increasing pore size and sediment permeability and fertilizer N uptake by rice. Therefore, the N content of rice also significantly increased in the groups with eels.

The emission of greenhouse gases, e.g. N_2_O and NH_3_, is one of the main methods of N loss from rice fields. In this study, the emission of N_2_O was significantly increased after the introduction of a high density of eels. Some behaviours of aquatic animals, e.g. digging burrows and foraging, can promote gas exchange among soil, water and atmosphere as well as enhance soil Eh, which contributes to the production of N_2_O through nitrification^30^. Moreover, the N substrates used for nitrification and denitrification can be obtained from the excretions of crayfish and eels. We found that NH_3_ volatilization was higher in the HD group than in the LD and control groups. This was possibly attributable to an increase in ionized ammonium (NH_4_^+^). Hargreaves considered that NH_3_ volatilization is determined by the equilibrium between unionized ammonia (NH_3_) and ionized ammonium (NH_4_^+^)^31^. All three groups showed a trend of increase in NH_3_ flux from June to September. This may be because the ingestion and excretion of crayfish and eels may be accelerated with an increase in temperature, thus increasing the concentrations of NH_3_ and NH_4_^+^.

In the mesocosm experiment, we found that the use efficiency of fertilizer N significantly improved with the introduction of eels in the RC system. However, the proportion of fertilizer N in the crayfish was significantly lower in the RCE system than in the RC system because the feeding habitat of crayfish may have been affected by the introduction of eels. A previous study indicated that about 58.6–65.3% of crayfish diets originated from aquatic plants, zooplankton and organic debris in the RC co-culture system^15^. However, the eel activities restricted the crayfish to the benthic zone, thus greatly reducing the probability of the crayfish of feeding on plants. Therefore, the N of crayfish in the RCE culture was mainly derived from the artificial diet, although no significant differences in total N were found between the RC and RCE systems. Wan et al. reported that muscle quality can be significantly improved in integrated RC culture (when compared with crayfish monoculture in ponds) because the crayfish can ingest more plant fibre^32^. Therefore, the quality of crayfish may be indirectly degraded in the RCE system, although their yields did not decrease significantly. To sum up, the introduction of appropriate amount of eels into rice–crayfish system may improve the availability of nitrogen fertilizer without increasing nitrogen loss to the environment.

## Conclusions

This study demonstrated the possibility of co-culture of crayfish and eels in rice fields. The addition of eels at a low density can promote the rice yield, while maintaining crayfish yield and N content in the environment. However, an overabundance of eels can cause a decline in crayfish yield. Moreover, total N content of the water and N_2_O emission increased significantly after the introduction of eels at a high density. More fertilizer N was used by rice and less N entered the crayfish from the fertilizer in the RCE system than in the RC system. The recycling of N in the field shows that the availability of fertilizer in the RC system can be effectively improved after the introduction of an appropriate number of eels.

## Materials and methods

### Field investigation

This study was performed between from May 2017 and October 2019 at Xinsheng Aquaculture Professional Cooperative (121° 0′ 56″ N, 30° 58′ 17″ E) in Qingpu District, Shanghai, Eastern China. This region has a subtropical monsoon climate with a mean monthly air temperature of 17.6 ± 2.3 °C and mean monthly precipitation of 126.9 ± 24.6 mm.

**Figure 4:**
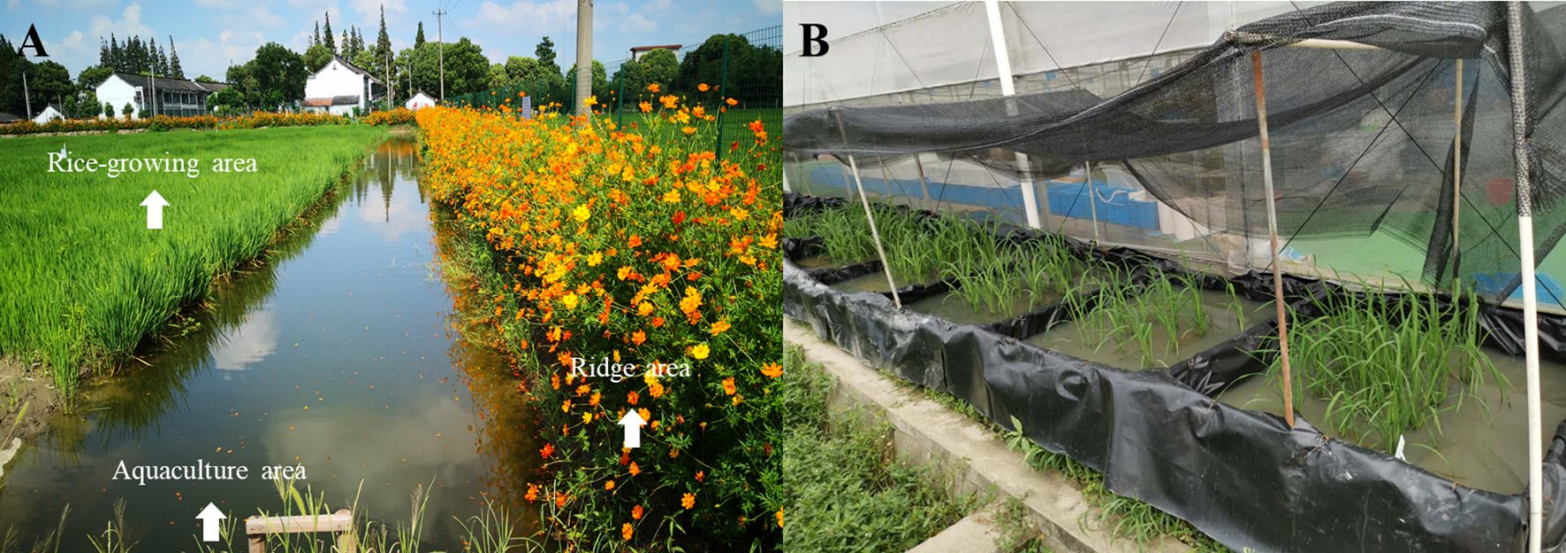
Photographs of the paddies in the field investigation (**A**) and plots in the mesocosm experiment (**B**).

Each RC paddy (667 m^2^) had a rice-growing area (80% of the total area), aquaculture area (10%) and ridge area (10%; Fig. 4A). In the aquaculture area, a 1.2 m deep ditch was dug to provide a more comfortable habitat for the crayfish and eels. The ridge had a height of 40 cm, and it was covered with a high-density polyethylene film to prevent the aquatic animals from escaping. Every May, rice (*Oryza sativa* L., Qing-Xiang-Ruan-Geng) seedlings were transplanted from a nursery into the paddies at a planting density of 20 × 20 cm (one seedling on each hill). Moreover, the juvenile crayfish weighing 1.5 ± 0.3 g were released into the paddies according to the standard of 45,000 juveniles per hectare, and the crayfish were allowed to self-propagate inside the rice paddies. A total of nine RC paddies were divided into three groups according to the rearing density of the eels: control group (C), low-density group (LD) and high-density group (HD) with rearing densities of 0, 6000 and 12,000 ind. ha^−1^, respectively. The LD and HD groups were supplemented with juvenile eels at a density of 2000 and 4000 ind. ha^−1^ in June 2018 and 2019. The average weights of juvenile eels in 2017, 2018 and 2019 were 21.4 ± 1.8, 24.1 ± 0.9 and 26.8 ± 1.1 g, respectively. All juvenile crayfish and eels were purchased from Shanghai Xiangsheng Aquaculture Cooperative. In the aquaculture area, floating plants, such as duckweed (*Lemna minor* L.) and foxtail (*Myriophyllum spicatum* L.), covered one-third of the water surface. The soil contained 20.6–23.7 g kg^−1^ of organic matter, 0.7–1.2 g kg^−1^ of total N and 0.31–0.37 g kg^−1^ of total P.

Only basal fertilizer was used for rice cultivation, and it contained 587 kg ha^−1^ of urea (46.4% N), 625 kg ha^−1^ of superphosphate and 150 kg ha^−1^ of potassium chloride. Every day, 500 g of commercial fish diet (5.83% N) was applied, and no pesticides or herbicides were used in the paddies.

In late August, the mature crayfish and eels were collected using ground cages to measure the aquatic product yields. The immature crayfish and eels were returned to the paddy fields during the collection. After the rice was harvested, the rice grains were air-dried and weighed to estimate the rice yield. The N content of the rice grains and aquatic animals was determined using the semi-micro Kjeldahl method^33^. Before testing, rice grains, crayfish and eels were weighted, dried at 65 °C and ground. Then, all the samples were digested with concentrated sulphuric acid (H_2_SO_4_) and hydrogen peroxide.

Water samples were collected every month during the co-culture period. Three duplicate 500 mL water samples were collected from 0 to 10 cm below the surface in the aquaculture area; the three subsamples were combined to obtain one sample per paddy. In the laboratory, the total N content of the water was analysed using UV spectrophotometry after digestion by alkaline potassium persulfate oxidation.

Soil samples were collected after the rice-planting period. In each paddy, three samples were collected from a rice-planting area of 0.25 m × 0.25 m × 0.10 m. All the soil samples were air-dried, ground, passed through a 0.15 mm sieve and digested with K_2_SO_4_–CuSO_4_–Se solution. Then, the semi-micro Kjeldahl method was used to test the total N content of the soil.

The N_2_O flux rate was measured using the static chamber method^34^. The size of the chamber was 1.0 m × 1.0 m × 1.0 m. The N_2_O samples were collected every half month between 8:30 and 10:30 AM from June to October. In each paddy, four gas samples were collected using 40 mL vacuum tubes at 10 min intervals (0, 10, 20 and 30 min after chamber closure). All samples were analysed with gas chromatography (GC 2010; Shimadzu, Kyoto, Japan). The N_2_O flux rate was calculated using the following equation:

1 $$F = \rho \times h \times \left[ {{273}/\left( {{273} + T} \right)} \right] \times {\text{d}}C{\text{/d}}t$$

where *F* is the N_2_O flux rate (μg N m^−2^ h^−1^); *ρ*, density of N_2_O at the standard state (μg m^−3^); *h*, height of the chamber (m); *T*, average temperature in the chamber during gas collection and d*C*/d*t*, concentration variation rate of N_2_O.

The ammonia volatilization flux was measured with a continuous airflow enclosure method^35^. The NH_3_ flux was measured every half month from 09:00 to 11:00 AM during the rice-planting period. NH_3_ was absorbed using boric acid, and 0.01 M H_2_SO_4_ was used to titrate the solution to determine the rate of NH_3_ volatilization. The ammonia volatilization flux was calculated using the following equation:

2 $$F = 14 \times V \times C \times A^{ - 1} \times t^{ - 1}$$

where *F* denotes the ammonia volatilization flux (mg N m^−2^ h^−1^); *V*, volume of H_2_SO_4_ titrated (L); *C*, concentration of H_2_SO_4_ (mol L^−1^); *A*, area of the chamber base (m^2^) and *t*, continuous measurement time.

In this study, all the data were shown as mean ± standard error of the mean (SEM) values. One-way ANOVA and Tukey’s test (SPSS V.16.0) were used to compare the differences of the yields and total N content among the three groups and three investigated years.

### Mesocosm experiment

Between May and October 2019, the mesocosm experiment was conducted at Shanghai Academy of Agricultural Sciences. Each mesocosm consisted of an experimental plot (1.2 m × 1.2 m × 0.6 m) covered with a high-density polyethylene film (Fig. 4B). In each experiment plot, 30 kg of soil from Xinsheng Aquaculture Professional Cooperative was used to construct a rice-planting platform and an aquaculture ditch (40 cm in depth). The platform area was about three-fourth of the cross-sectional area of the plot.

A total of six mesocosms were constructed: three experimental plots (RCE) and three control plots (RC). In each plot, the rice seedlings were planted in hills (one seedling per hill) within rows in May, with 20 cm between rows and 20 cm between hills in the same row for the experimental and control plots. The fertilizers used in each plot contained 84.5 g of urea (N content, 46.8%; ^15^N abundance, 10.15%), 90 g of superphosphate and 15 g of potassium chloride. The duckweed was planted in the aquaculture area, and it covered 30% of the aquaculture zone. Mudsnails (*Cipangopaludina cathayensis*, 500 g) were added to each plot. After a month, 12 crayfish were cultured in each simulated paddy, and two eels were reared in each experiment plot. The proportion of crayfish and eels was set according to that in the LD group of field investigation. The crayfish feed was supplied once every day, and the daily allowance was about 3% of the estimated crayfish weight in each mesocosm. The rice and aquatic products were harvested in October.

Rice, crayfish and eel samples were collected to measure the total N content and ^15^N abundance. The total N content of the soil and organism samples were measured using the semi-micro Kjeldahl method after digestion with concentrated H_2_SO_4_ and hydrogen peroxide. The ^15^N abundance was measured in all samples by using the MAT-271 isotope mass spectrometer (Finnigan MAT, California). The accumulation of N in rice, crayfish and eels from N fertilizer was calculated using the following equations:

3 $${\text{Percentage of accumulated N from fertilizer NDFF}})\;( \% ) = {\text{A}}\% \;{\text{E of the organism sample/A}}\% \;{\text{E of the fertilizer sample}} \times 100$$

4 $${\text{Amount of accumulated N from fertilizer}} = {\text{organism N accumulation amount}} \times {\text{NDFF}}$$

5 $${\text{N use efficiency NUE}})\;(\% ) = {\text{amount of N accumulated by the organism accumulated from N fertilizer/total N content of the fertilizer}} \times 100$$

where A% E is the difference between the ^15^N abundance of the samples or ^15^N-labelled fertilizers and natural abundance of ^15^N.

The independent-samples *t*-test was used to determine the differences in total N, N use efficiency and percentage of N derived from fertilizer between RCE and RC at 95% confidence level by using SPSS 16.0 (*P* value < 0.05 was considered statistically significant).

## Supplementary information

Supplementary Tables.
