# Supplementary material for: Effects of introducing eels on the yields and availability of fertilizer nitrogen in an integrated rice–crayfish system
Source: Sci Rep. 2020 Sep 9;10:14818. doi: 10.1038/s41598-020-71884-0 (PMC7481215; doi:10.1038/s41598-020-71884-0)
Supplement: Supplementary file 1 — Supplementary Tables. [file 41598_2020_71884_MOESM1_ESM.docx]

**Supplementary materials**

**Effects of introducing eels on the yields and availability of fertilizer nitrogen in an integrated rice–crayfish system**

Weiwei Lv^a^ , Quan Yuan^a^, Weiguang Lv^a^, Wenzong Zhou^a^*

*^a^* Eco-environmental Protection Research Institute, Shanghai Academy of Agricultural Sciences, Shanghai 201403, China

^*^ Corresponding author:

Wenzong Zhou, E-mail address: wzzhou505@sina.com

**Table S1.** One-way ANOVA in yields and total N content of rice, crayfish and eels among three groups from 2017 to 2019. The abbreviation represented different groups and years: C, control group; LD, low-density group; HD, high-density group; 17, 2017; 18, 2018; 19, 2019.

| Items |  |  | Analysis method | Source of variation | Yields | | | Total N | | |
| --- | --- | --- | --- | --- | --- | --- | --- | --- | --- | --- |
|  |  |  |  |  | DF | *F* | *P* | DF | *F* | *P* |
| Rice | Among years | C | One-way ANOVA |  | 2 | 9.9 | 0.002 | 2 | 3.8 | 0.085 |
|  |  |  | Tukey’s test | 17 vs. 18 |  |  | 0.013 |  |  | 0.231 |
|  |  |  |  | 17 vs. 19 |  |  | 0.002 |  |  | 0.079 |
|  |  |  |  | 18 vs. 19 |  |  | 0.607 |  |  | 0.692 |
|  |  | LD | One-way ANOVA |  | 2 | 0.8 | 0.483 | 2 | 0.5 | 0.640 |
|  |  |  | Tukey’s test | 17 vs. 18 |  |  | 0.576 |  |  | 0.955 |
|  |  |  |  | 17 vs. 19 |  |  | 0.993 |  |  | 0.790 |
|  |  |  |  | 18 vs. 19 |  |  | 0.514 |  |  | 0.628 |
|  |  | HD | One-way ANOVA |  | 2 | 1.0 | 0.420 | 2 | 0.3 | 0.786 |
|  |  |  | Tukey’s test | 17 vs. 18 |  |  | 0.986 |  |  | 0.888 |
|  |  |  |  | 17 vs. 19 |  |  | 0.445 |  |  | 0.776 |
|  |  |  |  | 18 vs. 19 |  |  | 0.527 |  |  | 0.973 |
|  | Among groups | 2017 | One-way ANOVA |  | 2 | 1.7 | 0.254 | 2 | 7.5 | 0.006 |
|  |  |  | Tukey’s test | LD vs. C |  |  | 0.229 |  |  | 0.019 |
|  |  |  |  | HD vs. C |  |  | 0.687 |  |  | 0.008 |
|  |  |  |  | LD vs. HD |  |  | 0.597 |  |  | 0.889 |
|  |  | 2018 | One-way ANOVA |  | 2 | 13.8 | <0.001 | 2 | 7.8 | 0.021 |
|  |  |  | Tukey’s test | LD vs. C |  |  | 0.003 |  |  | 0.023 |
|  |  |  |  | HD vs. C |  |  | <0.001 |  |  | 0.043 |
|  |  |  |  | LD vs. HD |  |  | 0.639 |  |  | 0.775 |
|  |  | 2019 | One-way ANOVA |  | 2 | 14.0 | 0.005 | 2 | 8.6 | 0.003 |
|  |  |  | Tukey’s test | LD vs. C |  |  | 0.012 |  |  | 0.004 |
|  |  |  |  | HD vs. C |  |  | 0.007 |  |  | 0.015 |
|  |  |  |  | LD vs. HD |  |  | 0.835 |  |  | 0.800 |
| Crayfish | Among years | C | One-way ANOVA |  | 2 | 0.5 | 0.652 | 2 | 2.1 | 0.201 |
|  |  |  | Tukey’s test | 17 vs. 18 |  |  | 0.934 |  |  | 0.384 |
|  |  |  |  | 17 vs. 19 |  |  | 0.827 |  |  | 0.215 |
|  |  |  |  | 18 vs. 19 |  |  | 0.632 |  |  | 0.968 |
|  |  | LD | One-way ANOVA |  | 2 | 0.2 | 0.795 | 2 | 0.2 | 0.799 |
|  |  |  | Tukey’s test | 17 vs. 18 |  |  | 0.968 |  |  | 0.963 |
|  |  |  |  | 17 vs. 19 |  |  | 0.783 |  |  | 0.784 |
|  |  |  |  | 18 vs. 19 |  |  | 0.902 |  |  | 0.911 |
|  |  | HD | One-way ANOVA |  | 2 | 0.3 | 0.737 | 2 | 0.9 | 0.439 |
|  |  |  | Tukey’s test | 17 vs. 18 |  |  | 0.779 |  |  | 0.815 |
|  |  |  |  | 17 vs. 19 |  |  | 1.000 |  |  | 0.410 |
|  |  |  |  | 18 vs. 19 |  |  | 0.772 |  |  | 0.743 |
|  | Among groups | 2017 | One-way ANOVA |  | 2 | 8.3 | 0.019 | 2 | 5.2 | 0.013 |
|  |  |  | Tukey’s test | LD vs. C |  |  | 0.545 |  |  | 0.939 |
|  |  |  |  | HD vs. C |  |  | 0.046 |  |  | 0.018 |
|  |  |  |  | LD vs. HD |  |  | 0.018 |  |  | 0.038 |
|  |  | 2018 | One-way ANOVA |  | 2 | 12.9 | 0.001 | 2 | 4.7 | 0.019 |
|  |  |  | Tukey’s test | LD vs. C |  |  | 0.927 |  |  | 0.834 |
|  |  |  |  | HD vs. C |  |  | 0.002 |  |  | 0.021 |
|  |  |  |  | LD vs. HD |  |  | 0.001 |  |  | 0.042 |
|  |  | 2019 | One-way ANOVA |  | 2 | 15.2 | <0.001 | 2 | 8.5 | 0.003 |
|  |  |  | Tukey’s test | LD vs. C |  |  | 0.197 |  |  | 0.904 |
|  |  |  |  | HD vs. C |  |  | 0.007 |  |  | 0.005 |
|  |  |  |  | LD vs. HD |  |  | <0.001 |  |  | 0.012 |
| Eel | Among years | C | One-way ANOVA |  | - | - | - | - | - | - |
|  |  |  | Tukey’s test | 17 vs. 18 |  |  | - |  |  | - |
|  |  |  |  | 17 vs. 19 |  |  | - |  |  | - |
|  |  |  |  | 18 vs. 19 |  |  | - |  |  | - |
|  |  | LD | One-way ANOVA |  |  |  |  |  |  |  |
|  |  |  | Tukey’s test | 17 vs. 18 |  |  | 0.951 |  |  | 0.975 |
|  |  |  |  | 17 vs. 19 |  |  | 0.760 |  |  | 0.739 |
|  |  |  |  | 18 vs. 19 |  |  | 0.910 |  |  | 0.853 |
|  |  | HD | One-way ANOVA |  | 2 | 1.9 | 0.231 | 2 | 1.4 | 0.310 |
|  |  |  | Tukey’s test | 17 vs. 18 |  |  | 0.620 |  |  | 0.718 |
|  |  |  |  | 17 vs. 19 |  |  | 0.207 |  |  | 0.283 |
|  |  |  |  | 18 vs. 19 |  |  | 0.620 |  |  | 0.664 |
|  | Among groups | 2017 | One-way ANOVA |  | 2 | 29.9 | 0.001 | 2 | 24.9 | 0.001 |
|  |  |  | Tukey’s test | LD vs. C |  |  | 0.036 |  |  | 0.082 |
|  |  |  |  | HD vs. C |  |  | 0.001 |  |  | 0.001 |
|  |  |  |  | LD vs. HD |  |  | 0.011 |  |  | 0.012 |
|  |  | 2018 | One-way ANOVA |  | 2 | 34.8 | <0.001 | 2 | 49.7 | <0.001 |
|  |  |  | Tukey’s test | LD vs. C |  |  | 0.041 |  |  | 0.035 |
|  |  |  |  | HD vs. C |  |  | <0.001 |  |  | <0.001 |
|  |  |  |  | LD vs. HD |  |  | 0.006 |  |  | 0.002 |
|  |  | 2019 | One-way ANOVA |  | 2 | 39.0 | <0.001 | 2 | 32.5 | 0.001 |
|  |  |  | Tukey’s test | LD vs. C |  |  | 0.040 |  |  | 0.084 |
|  |  |  |  | HD vs. C |  |  | <0.001 |  |  | 0.001 |
|  |  |  |  | LD vs. HD |  |  | 0.004 |  |  | 0.005 |

**Table S2.** One-way ANOVA in total N content of water and soils among three groups from 2017 to 2019. The abbreviation represented different groups and years: C, control group; LD, low-density group; HD, high-density group; 17, 2017; 18, 2018; 19, 2019.

| Items |  |  | Analysis method | Source of variation | Water | | | Soils | | |
| --- | --- | --- | --- | --- | --- | --- | --- | --- | --- | --- |
|  |  |  |  |  | DF | *F* | *P* | DF | *F* | *P* |
| Rice | Among years | C | One-way ANOVA |  | 2 | 1.4 | 0.323 | 2 | 0.4 | 0.710 |
|  |  |  | Tukey’s test | 17 vs. 18 |  |  | 0.396 |  |  | 0.788 |
|  |  |  |  | 17 vs. 19 |  |  | 0.370 |  |  | 0.992 |
|  |  |  |  | 18 vs. 19 |  |  | 0.998 |  |  | 0.722 |
|  |  | LD | One-way ANOVA |  | 2 | 9.6 | 0.094 | 2 | 0.2 | 0.817 |
|  |  |  | Tukey’s test | 17 vs. 18 |  |  | 0.750 |  |  | 0.954 |
|  |  |  |  | 17 vs. 19 |  |  | 0.150 |  |  | 0.802 |
|  |  |  |  | 18 vs. 19 |  |  | 0.545 |  |  | 0.934 |
|  |  | HD | One-way ANOVA |  | 2 | 3.2 | 0.115 | 2 | 0.6 | 0.599 |
|  |  |  | Tukey’s test | 17 vs. 18 |  |  | 0.163 |  |  | 0.891 |
|  |  |  |  | 17 vs. 19 |  |  | 0.145 |  |  | 0.574 |
|  |  |  |  | 18 vs. 19 |  |  | 0.995 |  |  | 0.829 |
|  | Among groups | June | One-way ANOVA |  | 2 | 4.7 | 0.058 |  |  |  |
|  |  |  | Tukey’s test | LD vs. C |  |  | 0.108 |  |  |  |
|  |  |  |  | HD vs. C |  |  | 0.066 |  |  |  |
|  |  |  |  | LD vs. HD |  |  | 0.921 |  |  |  |
|  |  | July | One-way ANOVA |  | 2 | 3.7 | 0.091 |  |  |  |
|  |  |  | Tukey’s test | LD vs. C |  |  | 0.381 |  |  |  |
|  |  |  |  | HD vs. C |  |  | 0.079 |  |  |  |
|  |  |  |  | LD vs. HD |  |  | 0.463 |  |  |  |
|  |  | August | One-way ANOVA |  | 2 | 9.1 | 0.015 |  |  |  |
|  |  |  | Tukey’s test | LD vs. C |  |  | 0.031 |  |  |  |
|  |  |  |  | HD vs. C |  |  | 0.019 |  |  |  |
|  |  |  |  | LD vs. HD |  |  | 0.899 |  |  |  |
|  |  | September | One-way ANOVA |  | 2 | 18.3 | 0.003 |  |  |  |
|  |  |  | Tukey’s test | 17 vs. 18 |  |  | 0.058 |  |  |  |
|  |  |  |  | 17 vs. 19 |  |  | 0.002 |  |  |  |
|  |  |  |  | 18 vs. 19 |  |  | 0.058 |  |  |  |
|  |  | October | One-way ANOVA |  | 2 | 15.9 | 0.004 |  |  |  |
|  |  |  | Tukey’s test | 17 vs. 18 |  |  | 0.108 |  |  |  |
|  |  |  |  | 17 vs. 19 |  |  | 0.003 |  |  |  |
|  |  |  |  | 18 vs. 19 |  |  | 0.054 |  |  |  |

**Table S3.** One-way ANOVA in emission of N_2_O and NH_3_ among three groups from 2017 to 2019. The abbreviation represented different groups and years: C, control group; LD, low-density group; HD, high-density group; 17, 2017; 18, 2018; 19, 2019.

| Items |  |  | Analysis method | Source of variation | N_2_O | | | NH_3_ | | |
| --- | --- | --- | --- | --- | --- | --- | --- | --- | --- | --- |
|  |  |  |  |  | DF | *F* | *P* | DF | *F* | *P* |
| Rice | Among years | C | One-way ANOVA |  | 2 | 2.5 | 0.165 | 2 | 1.2 | 0.374 |
|  |  |  | Tukey’s test | 17 vs. 18 |  |  | 0.366 |  |  | 0.347 |
|  |  |  |  | 17 vs. 19 |  |  | 0.769 |  |  | 0.794 |
|  |  |  |  | 18 vs. 19 |  |  | 0.154 |  |  | 0.681 |
|  |  | LD | One-way ANOVA |  | 2 | 0.3 | 0.729 | 2 | 3.6 | 0.095 |
|  |  |  | Tukey’s test | 17 vs. 18 |  |  | 0.798 |  |  | 0.132 |
|  |  |  |  | 17 vs. 19 |  |  | 0.994 |  |  | 0.125 |
|  |  |  |  | 18 vs. 19 |  |  | 0.743 |  |  | 0.999 |
|  |  | HD | One-way ANOVA |  | 2 | 0.6 | 0.595 | 2 | 0.7 | 0.538 |
|  |  |  | Tukey’s test | 17 vs. 18 |  |  | 0.832 |  |  | 0.947 |
|  |  |  |  | 17 vs. 19 |  |  | 0.885 |  |  | 0.528 |
|  |  |  |  | 18 vs. 19 |  |  | 0.568 |  |  | 0.704 |
|  | Among groups | June | One-way ANOVA |  | 2 | 1.3 | 0.351 | 2 | 3.0 | 0.125 |
|  |  |  | Tukey’s test | LD vs. C |  |  | 1.000 |  |  | 0.109 |
|  |  |  |  | HD vs. C |  |  | 0.409 |  |  | 0.522 |
|  |  |  |  | LD vs. HD |  |  | 0.413 |  |  | 0.445 |
|  |  | July | One-way ANOVA |  | 2 | 5.9 | 0.089 | 2 | 5.4 | 0.056 |
|  |  |  | Tukey’s test | LD vs. C |  |  | 0.269 |  |  | 0.936 |
|  |  |  |  | HD vs. C |  |  | 0.281 |  |  | 0.084 |
|  |  |  |  | LD vs. HD |  |  | 0.052 |  |  | 0.054 |
|  |  | August | One-way ANOVA |  | 2 | 4.6 | 0.040 | 2 | 2.6 | 0.155 |
|  |  |  | Tukey’s test | LD vs. C |  |  | 1.000 |  |  | 0.770 |
|  |  |  |  | HD vs. C |  |  | 0.045 |  |  | 0.145 |
|  |  |  |  | LD vs. HD |  |  | 0.046 |  |  | 0.348 |
|  |  | September | One-way ANOVA |  | 2 | 2.2 | 0.195 | 2 | 6.1 | 0.011 |
|  |  |  | Tukey’s test | 17 vs. 18 |  |  | 0.570 |  |  | 0.400 |
|  |  |  |  | 17 vs. 19 |  |  | 0.174 |  |  | 0.009 |
|  |  |  |  | 18 vs. 19 |  |  | 0.590 |  |  | 0.116 |
|  |  | October | One-way ANOVA |  | 2 | 30.3 | 0.001 | 2 | 10.5 | 0.001 |
|  |  |  | Tukey’s test | 17 vs. 18 |  |  | 0.713 |  |  | 0.672 |
|  |  |  |  | 17 vs. 19 |  |  | 0.001 |  |  | 0.009 |
|  |  |  |  | 18 vs. 19 |  |  | 0.002 |  |  | 0.002 |
